# Systemic Inhibition of Canonical Notch Signaling Results in Sustained Callus Inflammation and Alters Multiple Phases of Fracture Healing

**DOI:** 10.1371/journal.pone.0068726

**Published:** 2013-07-03

**Authors:** Michael I. Dishowitz, Patricia L. Mutyaba, Joel D. Takacs, Andrew M. Barr, Julie B. Engiles, Jaimo Ahn, Kurt D. Hankenson

**Affiliations:** 1 Department of Bioengineering, School of Engineering and Applied Sciences, University of Pennsylvania, Philadelphia, Pennsylvania, United States of America; 2 Department of Clinical Studies-New Bolton Center, School of Veterinary Medicine, University of Pennsylvania, Philadelphia, Pennsylvania, United States of America; 3 Department of Pathobiology, School of Veterinary Medicine, University of Pennsylvania, Philadelphia, Pennsylvania, United States of America; 4 Department of Orthopaedic Surgery, Perelman School of Medicine, University of Pennsylvania, Philadelphia, Pennsylvania, United States of America; The University of Adelaide, Australia

## Abstract

The Notch signaling pathway is an important regulator of embryological bone development, and many aspects of development are recapitulated during bone repair. We have previously reported that Notch signaling components are upregulated during bone fracture healing. However, the significance of the Notch pathway in bone regeneration has not been described. Therefore, the objective of this study was to determine the importance of Notch signaling in regulating bone fracture healing by using a temporally controlled inducible transgenic mouse model (Mx1-Cre;dnMAML^f/-^) to impair RBPjκ-mediated canonical Notch signaling. The Mx1 promoter was synthetically activated resulting in temporally regulated systemic dnMAML expression just prior to creation of bilateral tibial fractures. This allowed for mice to undergo unaltered embryological and post-natal skeletal development. Results showed that systemic Notch inhibition prolonged expression of inflammatory cytokines and neutrophil cell inflammation, and reduced the proportion of cartilage formation within the callus at 10 days-post-fracture (dpf) Notch inhibition did not affect early bone formation at 10dpf, but significantly altered bone maturation and remodeling at 20dpf. Increased bone volume fraction in dnMAML fractures, which was due to a moderate decrease in callus size with no change in bone mass, coincided with increased trabecular thickness but decreased connectivity density, indicating that patterning of bone was altered. Notch inhibition decreased total osteogenic cell density, which was comprised of more osteocytes rather than osteoblasts. dnMAML also decreased osteoclast density, suggesting that osteoclast activity may also be important for altered fracture healing. It is likely that systemic Notch inhibition had both direct effects within cell types as well as indirect effects initiated by temporally upstream events in the fracture healing cascade. Surprisingly, Notch inhibition did not alter cell proliferation. In conclusion, our results demonstrate that the Notch signaling pathway is required for the proper temporal progression of events required for successful bone fracture healing.

## Introduction

Bone fracture healing occurs through a series of carefully regulated spatiotemporal events. Following injury, hematoma formation and inflammation mediate an influx of undifferentiated mesenchymal cells to the site of injury. During endochondral fracture healing, these cells undergo chondrogenesis to produce a cartilaginous callus that mineralizes and is resorbed permitting vascular invasion of the callus. The vascular network mediates an influx of osteoprogenitor cells that differentiate to produce immature bone on top of the resorbing cartilage matrix. Callus bone matures and is remodeled over time through osteoblast-mediated bone formation and osteoclast-mediated bone resorption [1].

Bone fractures are a significant clinical and economic problem. While the majority of fractures restore original structure and function in a scarless manner, some fractures result in delayed or non-union healing [2]. This increases the cost of care, necessitates additional surgeries, and results in a prolonged period of convalescence, which is associated with increased mortality in an aged population [3]. Common therapeutic strategies such as autologous bone grafts and bone morphogenetic proteins have well-documented limitations [4,5]. Therefore, a clinical need persists for the development of new methods to enhance healing. Although the spatiotemporal progression of fracture healing is well-characterized [1], the signaling pathways that regulate events required for healing are not as well understood. Identifying and elucidating the roles of signaling pathways that regulate fracture healing will allow us to identify novel therapeutic targets for improved regeneration of bone.

Notch signaling—which has been implicated in bone formation and fracture healing—is a developmentally conserved pathway that regulates cell proliferation and differentiation [6]. Activation of the cell-to-cell signaling pathway occurs when a Notch ligand (Jagged 1,2 and Delta-like 1,4) expressed on the surface of a signaling cell interacts with a Notch receptor (Notch 1-4) expressed on the surface of a receiving cell. A two-stage proteolytic event liberates the Notch intracellular domain (NICD), which translocates to the nucleus and binds to the Recombination Signal Binding Protein For Immunoglobulin Kappa J Region (RBPjκ) and Mastermind-like proteins (MAML). MAML serves as a scaffold to recruit other co-activators required to initiate transcription of canonical Notch target gene families Hes and Hey.

Importantly, the Notch signaling pathway regulates multiple cell lineages that participate in bone formation. Notch upregulation in mesenchymal progenitor cells promotes proliferation while inhibiting differentiation [7,8]. In committed chondroprogenitors, Notch inhibition promotes differentiation but is reactivated for terminal hypertrophic maturation [8–12]. In committed osteoprogenitors, Notch inhibition also promotes differentiation [7,13]. However, Notch components are endogenously expressed at various stages of maturation [11], where expression in mature osteoblasts and osteocytes indirectly inhibits osteoclast differentiation [7,13–15]. Notch signaling also inhibits osteoclast differentiation directly through expression in macrophage precursors [16]. These studies have collectively demonstrated that the Notch signaling pathway regulates embryological bone development in a temporally-sensitive and cell-context development manner.

Bone fracture healing recapitulates many aspects of embryological bone development [17–19], and we’ve previously shown that Notch signaling is active in mesenchymal lineages during fracture healing [11]. Furthermore, Notch signaling has also been shown to regulate tissue repair of other injuries [20]. Collectively, the data suggests that Notch signaling may also regulate bone fracture healing. Therefore, the objective of this study was to determine the importance of Notch signaling in regulating bone fracture healing by using a temporally controlled inducible transgenic mouse model to impair RBPjκ-mediated canonical Notch signaling in all cells during repair. Herein, we show that systemic inhibition of canonical Notch signaling alters multiple phases of the fracture healing process.

## Methods

### Generation of mice

Dominant negative MAML (dnMAML – Mx1-Cre+; dnMAML^f/-^) and wild type (WT – Mx1-Cre-; dnMAML^f/-^) mice generated on a C57BL/6 background were utilized in this study. The GFP-dnMAML fusion transgene is a truncated version of MAML, and contains the NICD domain that allows it to bind to the NICD-RBPjκ complex, but lacks the domain necessary to recruit other co-activators required to initiate transcription of target genes. Therefore, dnMAML inhibits canonical Notch signaling at the level of transcriptional complex assembly just prior to gene transcription [21,22]. The dnMAML-GFP transgene is preceded by a transcriptional stop sequence flanked by LoxP sites allowing it to be conditionally regulated by Cre recombinase expression [23–25]. The inducible Mx1-Cre promoter was used in this study to systemically activate dnMAML expression just prior to fracture [26], which allowed all mice to undergo unaltered embryological development and skeletal maturation. The normally silent Mx1 promoter can be induced by intraperitoneal (IP) injection of polyinosinic-polycytidylic acid (poly I:C). Resulting expression of Cre recombinase deletes the upstream transcriptional stop sequence allowing for systemic dnMAML expression at the ROSA26 locus.

### Experimental Design

All *in vivo* protocols were approved by the Institutional Animal Care and Use Committee of the University of Pennsylvania. At the onset of skeletal maturity at 3 months of age [27,28], dnMAML and WT mice were IP injected with 500 µg of poly I:C 10 times over 20 days. Allowing mice to undergo unaltered embryological bone development and skeletal maturation up to 3 months of age allowed the experiments to focus specifically on the role of Notch signaling during bone fracture healing, instead of during skeletal development. This protocol induces dnMAML-GFP expression in greater than 95% of total bone marrow cells [25] and 90% of bone marrow-derived mesenchymal progenitor cells [29].

After poly I:C injections, closed bilateral, transverse tibial fractures were created in the mid-diaphyseal region according to previously published methods. We used a custom-made three-point bending apparatus with intramedullary pin fixation of the tibia, resulting primarily in endochondral bone repair [11,30,31]. Radiographs verified correct pin placement and fracture generation (Faxitron X-Ray). 0.05 mg/kg of buprenorphine was administered subcutaneously twice daily for four days following injury, including a pre-operative dose. Mice recovered on heating pads and were allowed to ambulate freely. Fracture calluses were harvested for semi-quantitative real-time polymerase chain reaction (RT-PCR) analysis of gene expression at 5, 10 and 20 days-post-fracture (dpf) (n=6-9), quantitative histology and immunohistochemistry (IHC) at 10 and 20dpf (n=4-7), and micro-computed tomography (μCT) at 10 and 20dpf (n=7-13). The number of specimens analyzed per group, per time point, per assay is summarized in Figure S1. Mice were euthanized by CO_2_ exposure. Both males and females were included in this experiment to decrease the number of animals used. Several studies have reported similar responsivity of both sexes to manipulations of Notch signaling [7,14,32,33]. However, because male and female skeletons present with different quantities of bone during aging [27], the sexes were separated into different time points for histological and μCT analysis of bone and cartilage. Females were harvested at 10dpf, males at 20dpf, and mixed gender at 5dpf for gene expression analysis prior to bone or cartilage formation.

### Histology and IHC

Tissue was fixed in 4% paraformaldehyde at 4°C for 2-3 days, decalcified in 15% formic acid, paraffin embedded, and sectioned at 5 µm. For IHC, sections were deparaffinized and gradually rehydrated. Heat-mediated antigen retrieval via the microwave method was performed using Sodium Citrate Buffer at pH 6.0 for 20 minutes at 100% power (for Proliferating Cell Nuclear Antigen antibody) or Citra Plus (Biogenex) for 2 minutes at 100% followed by 15 minutes at 20% (for GFP antibody), and then cooled in buffer to room temperature. Sections were incubated in serum blocking solution (5% donkey serum, 4% BSA, 0.1% Triton-X 100, 0.05% Tween 20 in PBS) for 60 minutes, and then with primary antibody (see below) diluted in buffer solution (0.5% donkey serum, 2.4% BSA, 0.26% Triton-X 100, 0.005% Tween 20 in PBS) overnight at 4°C in a humidified chamber. Control sections were incubated in buffer solution only. Sections were then treated with 3% H_2_O_2_ for 30 minutes, followed by biotinylated secondary antibody Donkey anti-Rabbit (Santa Cruz sc-2089, 1:200 diluted in 0.5% donkey serum, 0.4% BSA, 0.01% Triton-X 100, 0.055% Tween 20 in PBS, 2 µg/ml working concentration) for 30 minutes, and finally streptavidin-HRP (Abcam ab7403, 1:500 diluted in PBS, 2 µg/ml working concentration) for 30 minutes. Sections were developed with 3,3’-Diaminobenzidine (DAB, Vector Laboratories) and counterstained with Hematoxylin. All incubations other than antigen retrieval and primary antibody were done at room temperature. Sections were washed in 0.02% Tween 20 in PBS after each step except between serum blocking and primary antibody incubation.

To identify cells that express the dnMAML-GFP transgene, sections were stained with Rabbit anti-GFP antibody (Abcam ab6556, 1:100, 5 µg/ml working concentration). To quantify cell proliferation, sections were stained with Rabbit anti-Proliferating Cell Nuclear Antigen (PCNA) antibody (Abcam ab2426, 1:100, 2 µg/ml working concentration), which is expressed in cells undergoing DNA synthesis. To quantify cartilage formation, sections were stained with Safranin O (SafO), which stains proteoglycans red, and a Fast Green counterstain. To quantify osseous tissue formation, sections were stained with Masson’s Trichrome (Sigma HT15-1KT), which stains collagenous tissue blue. Sections were also stained with Hematoxylin and Eosin (H&E) for semi-quantitative analysis of inflammation and a Gram stain for bacterial infection.

### Histomorphometric Analysis

Slides were imaged in bright field with an Olympus BX51. Color images were acquired with a Spot RT3 2 megapixel camera. ImageJ (National Institutes of Health) was used to quantify all histological data. All longitudinal histological sections analyzed were derived from the central portion of the callus where both cortices of the original bone were present. Two sections per specimen located on each half of the callus were analyzed.

20x SafO images of the entire longitudinal cross-sectional callus were acquired and stitched together as needed for analysis of cartilage formation. Contours were manually drawn around the total callus area excluding original cortical bone, marrow and muscle tissue. A fixed, global, color threshold was used for automated quantitation of cartilage area for all specimens. For 10dpf specimens, cartilage components were further broken down into immature, mature, and hypertrophic cartilage using semi-automated analysis based on cell morphology and intensity of SafO staining. 400x images were acquired in these areas for automated analysis of proliferating, pre-hypertrophic, and hypertrophic chondrocyte cell density.

20x Masson’s Trichrome images were similarly acquired for analysis of osseous bone tissue formation. Total callus area and osseous bone tissue area were similarly quantified. High-resolution images were acquired in areas of immature bone and mature bone for manual analysis of active osteoblast density (400x), osteocyte density (200x) and osteoclast density (200x). Active osteoblasts were defined as mononuclear cells aligning the bone surface with a cuboidal or columnar morphology. Osteocytes were defined as cells located within a lacunae surrounded by an osteoid or bony matrix. Osteoclasts were defined as cells aligning the bone surface with greater than two nuclei. Cell-based analysis at 10dpf incorporates areas of immature bone only, whereas cell-based analysis at 20dpf incorporates images taken in areas of both immature and mature bone.

400x PCNA images of 10dpf specimens were acquired in areas of undifferentiated mesenchymal cells and pre-hypertrophic chondrocytes for automated analysis of percent PCNA positive cells at each stage of differentiation. Similar analysis was conducted for area of PCNA staining in areas of immature bone.

For semi-quantitative analysis of inflammation at 10dpf, H&E sections were graded for neutrophil and mononuclear cell (macrophages and lymphocytes) inflammation individually. Presence of mast cells was ruled out by Toluidine blue staining. A score of 1-5 was given based on the level of inflammatory cell infiltration within each of the intramedullary cavity, the callus surrounding cortical bone, and the periosteal callus, and the scores were added together for a maximum of 15. For neutrophil inflammation, a score >12 indicated high inflammation (30-50%), 9-12 indicated micro abscess formation, 6-9 indicated moderate inflammation (10-30%), 3-6 indicated mild inflammation (<10%), and 3 indicated no inflammation. Similarly, for mononuclear cell inflammation, a score >12 indicated severe, 9-12 moderate, 6-9 mild, 3-6 minimal, and 3 no inflammation. Only one section per callus was analyzed for inflammation.

### Micro-Computed Tomography (μCT)

Tibial fracture calluses were scanned using a Scanco vivaCT40 (Scanco Medical) with the following parameters: 21 µm isotropic voxel size, 55 kVp. 145 µA, 500 projections per 180°, 650 millisecond integration time, 2D transverse reconstructed 1024x1024 pixel images. User-defined contours were drawn every 10 images (0.210 mm) or less around the callus for inclusion, with automated morphing used to interpolate the contours for all of the images in between. Similarly, user-defined contours were drawn around the original cortical bone and marrow cavity for exclusion with automated morphing in between. This semi-automated segmentation method analyzes the callus outside the pre-existing cortical bone. The entire length of the callus was analyzed. A fixed, global threshold of 16% of the maximum gray value, which corresponds to a mineral density of 169.8 mg HA/cm^3^ was applied to distinguish mineralized from unmineralized tissue. The following parameters were quantified: total callus volume, callus bone volume, bone volume fraction, tissue mineral density, trabecular number, trabecular thickness, trabecular separation, connectivity density, and structure model index.

### RT-PCR Analysis of Gene Expression

Fracture calluses were dissected from the surrounding tissue, placed in Qiazol lysis reagent (Qiagen) and stored at -80°C until further processing. Tissue was then homogenized using the Tissue Tearor (BioSpec Products) and mRNA was extracted using the Qiagen miRNeasy Mini Kit with DNase digestion to remove DNA contamination. RNA yield was determined using a NanoDrop 1000 spectrophotometer (ThermoScientific). 1 µg of mRNA was reverse transcribed into 20 µl of cDNA using the Applied Biosystems High Capacity RNA-to-cDNA Kit, and then diluted with RNase- and DNase-free H_2_O to a 40 µl volume. Gene expression was quantified using a 7500 Fast Real-Time PCR system (Applied Biosystems) from a total of 10 µl of Master Mix per well, which included 1x Fast SYBR Green (Applied Biosystems), forward and reverse primers (0.45 µM), and 0.5 µl of cDNA. For each gene of interest (Figure S2), samples were run in duplicate and control wells were run to rule out DNA contamination and primer dimer amplification. Proper amplicon formulation was confirmed by melt curve analysis. All RT-PCR data is presented as relative gene expression to β-actin housekeeping control, calculated using the formula 2^-ΔC(t)^, except for Notch target and GFP gene expression data which is presented as fold-change to WT control for each time point (5dpf dnMAML normalized to 5dpf WT, 10dpf dnMAML normalized to 10dpf WT, and 20dpf dnMAML normalized to 20dpf WT), calculated using the formula 2^-ΔΔC(t)^.

### Statistical Analysis

For parameters quantified at multiple time points, including gene expression (data normalized to β-actin), histology (20x), and μCT analysis, a two-way ANOVA was performed to test the main effects of dnMAML expression and time, and the interaction between the two. The main objective of this study is to evaluate how dnMAML expression affects fracture healing. Therefore, post-hoc Student’s t-tests were performed to compare dnMAML to WT at each time point only if there was a significant (*p<0.050) or trend ( ^t^p<0.100) effect of either dnMAML expression or the interaction between dnMAML expression and time.

For analysis specifically of Notch gene expression (data normalized to WT control for each time point), or for cell- and tissue-specific histomorphometric analysis (200x and 400x), a Student’s t-test was used to compare dnMAML to WT at each time point. For semi-quantitative analysis of neutrophil and mononuclear cell inflammation, a Mann-Whitney U non-parametric test was used to compare dnMAML to WT. Results of all statistical tests are summarized in Figure S3. Data is presented as mean ± standard deviation.

### Isolation and Culture of Murine Mesenchymal Stem Cells (mMSC)

Bone marrow cells were obtained from aseptically dissected uninjured femurs from poly I:C treated dnMAML (Mx1-Cre+; dnMAML^f/-^) and WT mice (Mx1-Cre-; dnMAML^f/-^) as previously published [34]. Whole marrow was expelled from the bone with several flushes through a 23-gauge needle. Single-cell suspensions were made by aspiration of the marrow through an 18-gauge needle for 5 minutes. Cells were pelleted, re-suspended, and grown in MSC media (αMEM, 10% fetal calf serum, 100x L-glutamine, 25 µg/mL ascorbic acid 2-phosphate, 100 IU/mL penicillin, 100 mg/mL streptomycin)

### Cell Number Assay

At the first or second passage, mMSCs were seeded at 2.6 x 10^4^ cells/cm^2^ into 12-well tissue culture plates and cultured in MSC media. At 24 and 96 hours, wells were incubated in a 10% alamarBlue solution (Invitrogen) diluted in MSC media for 2.5 hours. Cell number was fluorescently measured (excitation 570 nm, emission 585 nm), and wells were refreshed with new media. Three experiments were run with duplicate wells.

### Osteoblast Differentiation Assay

At the first or second passage, mMSCs were seeded at 2.6 x 10^4^ cells/cm^2^ into 12-well tissue culture plates and grown to confluence in MSC media, at which point they were cultured in osteogenic differentiation media (OGM: αMEM, 10% fetal calf serum, 100x L-glutamine, 100 IU/mL penicillin, 100 mg/mL streptomycin, 32.3 µg/mL ascorbic acid 2-phosphate, 5 mM β-glycerophosphate) for 2 weeks. Cells were then fixed in 70% ethanol for 10 minutes and incubated in 2% Alizarin red S (pH 4.25) for 10 minutes. Alizarin red S stains calcified mineral tissue red, which is indicative of mMSC terminal osteoblast differentiation. Excess Alizarin red S was removed and then calcium-bound Alizarin was extracted by treating with 10% (w/v) cetylpyridinium chloride in 10 mM/L sodium phosphate (pH 7.0). Total mineral content was then measured colorimetrically at 562 nm using a spectrophotometer. Four experiments were run with duplicate wells.

## Results

### dnMAML expression during bone fracture healing

**Figure 1:**
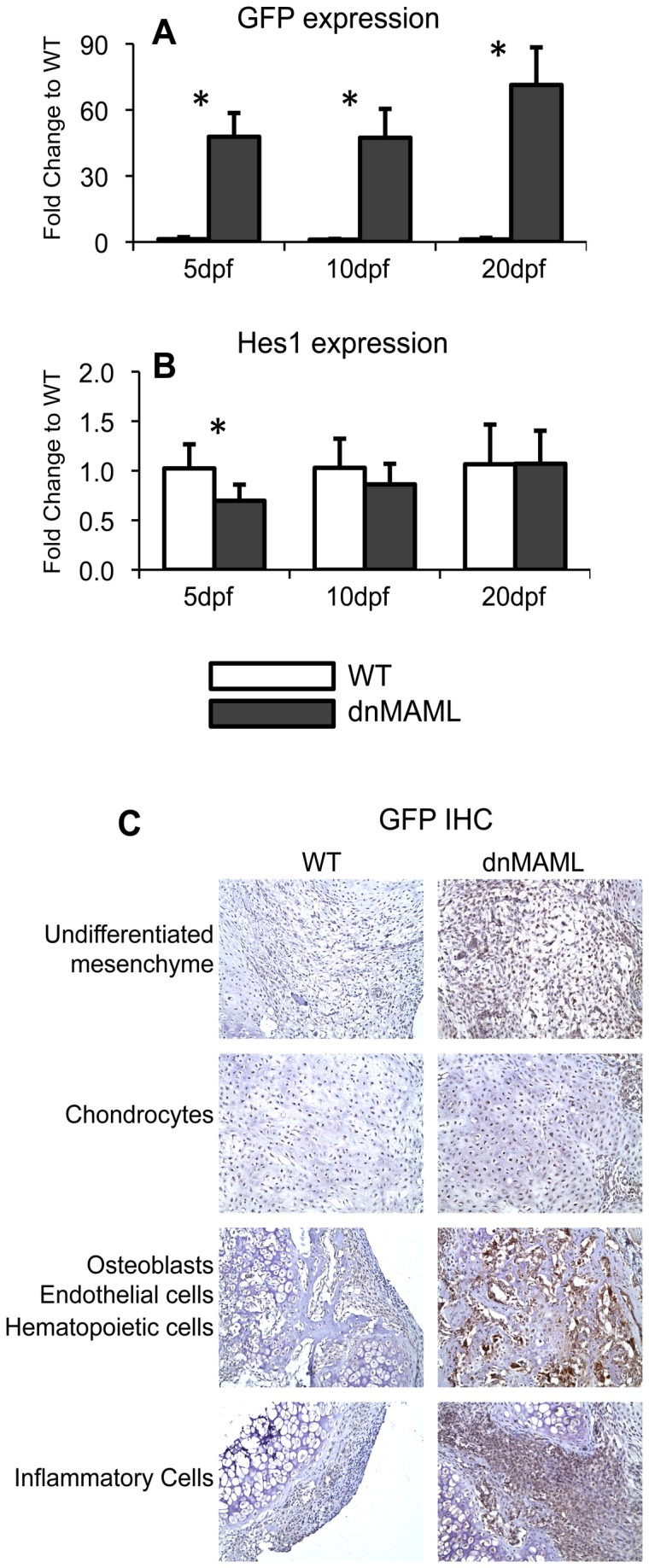
GFP-dnMAML fusion protein is expressed in dnMAML mice and inhibits Notch signaling during fracture healing. (**A**) GFP gene expression is upregulated in dnMAML fractures. (**B**) This correlates with a 30% reduction in Hes1 gene expression at 5dpf. (**C**) GFP is expressed in undifferentiated mesenchymal cells, chondrocytes, osteoblasts, endothelial cells, hematopoietic cells and inflammatory cells in dnMAML fractures. There is no expression in WT mice. GFP IHC images were acquired at 200x magnification. Gene expression data is presented as fold change to WT for each time point, calculated using the formula. 2^-ΔΔC(t)^. *p<0.050 (dnMAML vs WT).

The dnMAML transgene is a GFP fusion protein; thus, GFP can be used to assess dnMAML expression. GFP gene expression was upregulated 45-70 fold in dnMAML mice relative to WT mice at 5, 10 and 20dpf (Figure 1A). This corresponded to a 30% reduction in Hes1 gene expression at 5dpf (Figure 1B). GFP was also widely expressed in multiple cell populations present during fracture healing in dnMAML mice including undifferentiated mesenchymal cells, chondrocytes, osteoblasts, endothelial cells, hematopoietic cells, and inflammatory cells (Figure 1C), verifying that dnMAML was expressed during fracture healing. Expression was undetectable in WT mice.

### dnMAML decreases cartilage formation during fracture healing

**Figure 2:**
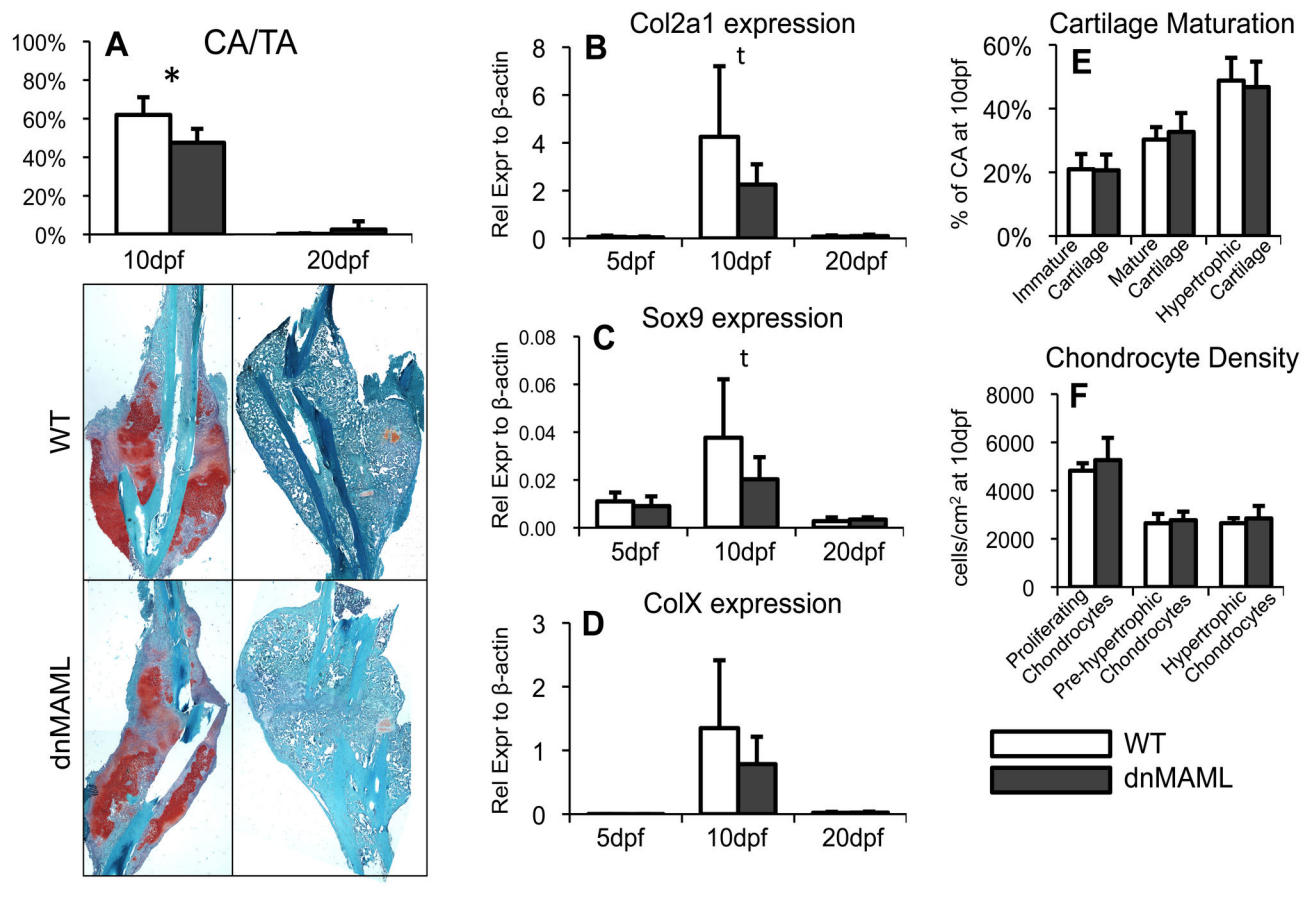
dnMAML decreases cartilage formation during fracture healing. (**A**) Percent of cartilage area to total callus area (CA/TA) via SafO histomorphometric analysis is decreased in dnMAML fractures at 10dpf. (**B**) Col2a1 and (**C**) Sox9 gene expression are decreased in dnMAML fractures at 10dpf. (**D**) ColX gene expression is non-significantly decreased in dnMAML fractures at 10dpf. (**E**) There are no differences between WT and dnMAML fractures in percent of immature, mature or hypertrophic cartilage to cartilage area (CA) at 10dpf. (**F**) There are no differences in chondrocyte density within these areas at 10dpf. SafO images were acquired at 20x magnification. Gene expression data is presented as relative expression to β-actin, calculated using the formula. 2^-ΔC(t)^. *p<0.050 ^t^p<0.100 (dnMAML vs WT).

During the endochondral phase of fracture healing, undifferentiated mesenchymal cells condense at the fracture site and undergo chondrogenesis to produce an initial cartilaginous callus matrix. To evaluate cartilage formation, sections were stained with SafO at 10 and 20dpf and chondrogenic gene expression was assessed at 5, 10 and 20dpf. dnMAML fractures had decreased percent cartilage area within the callus (CA/TA) at 10dpf (Figure 2A). Almost all cartilage was resorbed in both groups by 20dpf. Consistent with these histological results, dnMAML fractures had decreased Col2a1 (Figure 2B) and Sox9 (Figure 2C) gene expression at 10dpf, but were not different from WT at 5 or 20dpf. A two-way ANOVA showed decreased ColX gene expression in dnMAML fractures, though post-hoc analysis did not reveal time-point specific differences (Figure 2D). Collectively, the data demonstrates that dnMAML expression decreases cartilage formation during endochondral fracture healing.

The cartilage matrix initially comprised of immature cartilage populated by proliferating chondrocytes, develops first into mature cartilage populated by pre-hypertrophic chondrocytes and then finally into hypertrophic cartilage populated by hypertrophic chondrocytes. To evaluate differences in relative cartilage maturation, the specific components of the cartilage matrix were quantified based on maturity at 10dpf when peak formation occurs. There were no differences between dnMAML and WT mice in the percent of immature cartilage, mature cartilage, or hypertrophic cartilage to total cartilage area within the callus, demonstrating that the rate of cartilage maturation was not delayed or enhanced by dnMAML expression (Figure 2E). Chondrocyte density within each of these regions was also not affected by dnMAML expression, indicating that dnMAML expression did not affect individual chondrocyte function, specifically matrix production (Figure 2F).

### dnMAML alters bone remodeling during fracture healing

During endochondral fracture healing, immature bone is produced on top of a resorbing cartilage callus. Maturation and remodeling occurs over time through osteoblast-mediated bone formation and osteoclast-mediated bone resorption. To evaluate bone mass within the callus, fractures were analyzed via three-dimensional µCT and two-dimensional Masson’s Trichrome histology at 10 and 20dpf. Gene expression was also assessed at 5, 10 and 20dpf.

dnMAML expression appeared to have no affect on early bone formation (5, 10dpf). However, several molecular and phenotypic changes were observed during later stages of repair (20dpf), suggesting that dnMAML expression alters bone maturation and remodeling

**Figure 3:**
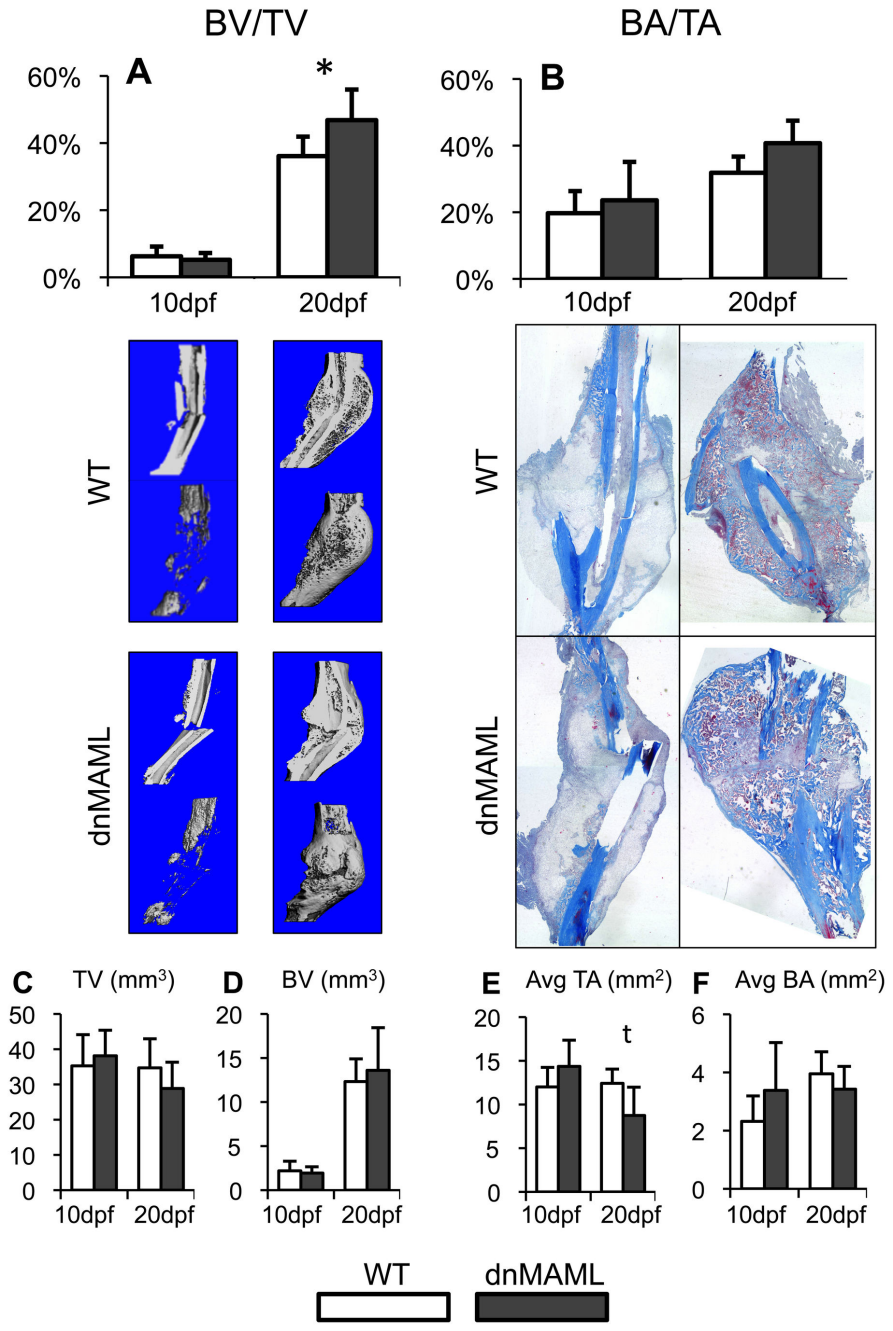
dnMAML increases the proportion of bone mass within the callus due to a moderate decrease in callus size. (**A**) Bone volume fraction (BV/TV) via μCT analysis is increased in dnMAML fractures at 20dpf. (**B**) There is a non-significant increase in percent osseous bone tissue area to total callus area (BA/TA) via Masson’s Trichrome histomorphometric analysis. (**C**) Callus total volume is non-significantly decreased and (**E**) average callus total area (Avg TA) is decreased at 20dpf. (**D**) There are no differences in bone volume (BV) or (**F**) average osseous bone tissue area (Avg BA) at any time point. μCT images were acquired at a 21 µm voxel size. Masson’s Trichrome images were acquired at 20x magnification. *p<0.050 ^t^p<0.100 (dnMAML vs WT).

Specifically, dnMAML expression increased the proportion of bone mass within the callus, indicated by increased bone volume fraction (BV/TV) (Figure 3A), and a non-significant increase in osseous bone tissue area within the callus (BA/TA) (Figure 3B). However, this appears to be the result from a moderate decrease in callus size, as measured by total volume (TV) and average total area (Avg TA), with no difference in total bone mass, as measured by bone volume (BV) and average osseous bone tissue area (Avg BA) (Figure 3C–F). dnMAML expression also increased trabecular thickness (Tb.Th), though there were no differences in trabecular number (Tb.N), separation (Tb. Sp) or tissue mineral density (TMD). Alternatively, structural model index (SMI) and trabecular connectivity density (Conn.D) were decreased (Table 1). Collectively, these results indicate that dnMAML expression increased the proportion of bone within the callus, but total amount was not changed, and the patterning of bone formation was significantly altered.

**Table 1:** μCT morphometric analysis of WT and dnMAML fractures at 10 and 20dpf.

	10dpf		20dpf	
	WT	dnMAML	WT	dnMAML
Tb.N (1/mm)	1.13 ± 0.19	1.05 ± 0.22	6.08 ± 0.43	6.37 ± 0.42
Tb.Th (mm)	0.110 ± 0.013	0.111 ± 0.017	0.099 ± 0.013	0.137 ± 0.033*
Tb. Sp (mm)	0.98 ± 0.13	1.05 ± 0.22	0.17 ± 0.02	0.16 ± 0.02
TMD (mg HA/cm^3^)	347 ± 19	344 ± 18	420 ± 22	438 ± 27
SMI	2.0 ± 0.4	2.2 ± 0.7	1.1 ± 0.7	- 0.3 ± 1.5*
Conn. D (1/mm^3^)	15.1 ± 10.9	9.9 ± 3.5	175 ± 19	150 ± 21*

Trabecular number (Tb.N), trabecular thickness (Tb.Th), trabecular separation (Tb. Sp), tissue mineral density (TMD), structural model index (SMI) and connectivity density (Conn.D) were quantified via μCT. Results are presented at mean ± standard deviation. *p<0.050 (WT vs dnMAML)

**Figure 4:**
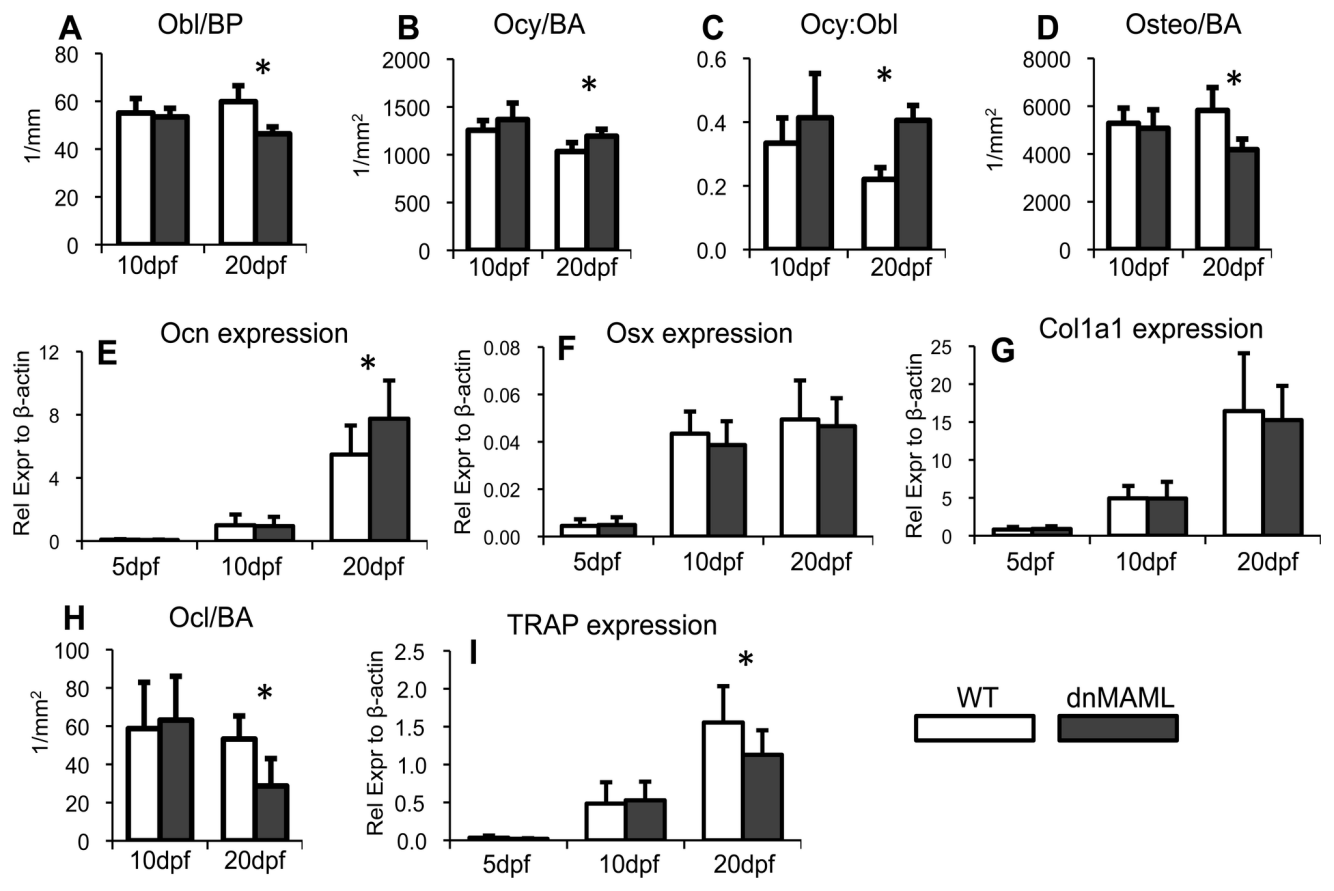
dnMAML decreases osteogenic and osteoclast cell density. (**A**) dnMAML expression decreases osteoblast density normalized by bone perimeter (Obl/BP) and (**B**) increases osteocyte density normalized by bone area (Ocy/BA) at 20dpf, (**C**) which results in an increased osteocyte-to-osteoblast ratio (Ocy:Obl). (**D**) However, dnMAML expression decreases overall osteogenic (osteoblast and osteocyte) cell density normalized by bone area (Osteo/BA) at 20dpf. (**E**) Osteocalcin (Ocn) gene expression is increased in dnMAML fractures at 20dpf. There are no differences in (**F**) osterix (Osx) or (**G**) collagen type I (Col1a1) gene expression. (**H**) dnMAML expression decreases osteoclast density normalized by bone area (Ocl/BA) and (**I**) TRAP gene expression at 20dpf. Masson’s Trichrome images were acquired at 200x and 400x magnification for cell-based histomorphometric analysis. Gene expression data is presented as relative expression to β-actin, calculated using the formula. 2^-ΔC(t)^. *p<0.050 (dnMAML vs WT).

To better understand the regulatory mechanism of altered bone modeling, osteoblast, osteocyte and osteoclast cell populations were characterized. dnMAML expression decreased osteoblast density (Obl/BP), yet increased osteocyte density (Ocy/BA), which resulted in an increased osteocyte-to-osteoblast ratio (Ocy:Obl) (Figure 4A–C). This correlated with an increase in osteocalcin (Ocn) gene expression, which is expressed by mature osteoblasts and osteocytes (Figure 4E). However, total osteogenic cell density (Osteo/BA), which includes both osteoblasts and osteocytes, was still decreased in dnMAML fractures (Figure 4D), which may explain why osterix (Osx) and collagen type I (Col1a1) gene expression were unchanged (Figure 4F,G). dnMAML expression also decreased osteoclast density (Ocl/BA), which correlated with a decrease in Tartrate-Resistant Acid Phosphatase (TRAP) gene expression (Figure 4H,I). In summary, dnMAML expression decreased the total number of osteogenic cells – which were proportionately more mature osteocytes – and decreased osteoclast number and gene expression.

### dnMAML prolongs inflammation during fracture healing

**Figure 5:**
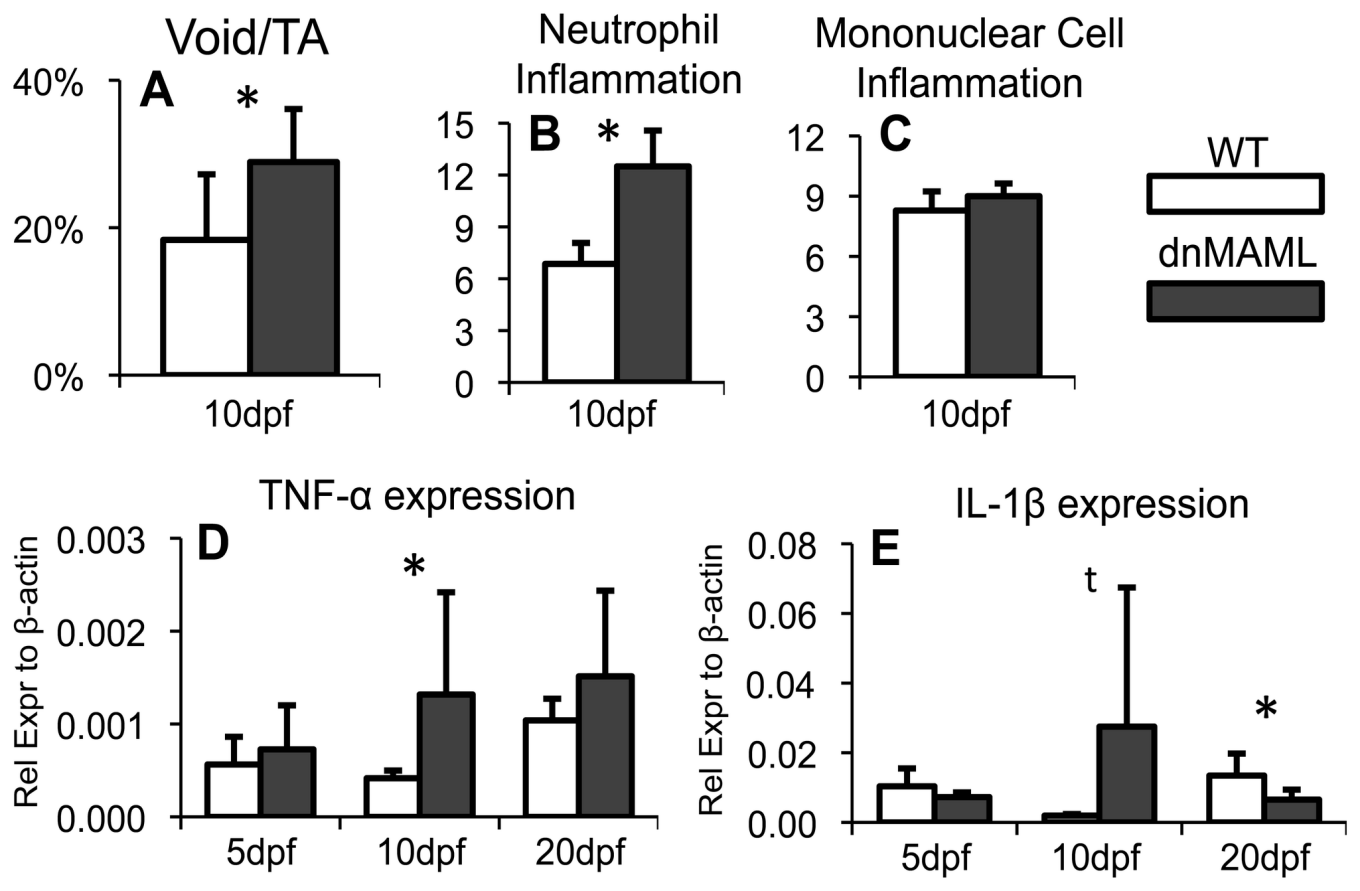
dnMAML prolongs inflammation during fracture healing. (**A**) Percent void area to total callus area (Void/TA) via histomorphometric analysis is increased in dnMAML fractures at 10dpf. (**B**) Neutrophil inflammation via semi-quantitative analysis of H&E images is increased in dnMAML fractures at 10dpf. (**C**) There is no difference in mononuclear cell inflammation. (**D**) TNF-α and (**E**) IL-1β gene expression are increased in dnMAML fractures at 10dpf. IL-1β is decreased at 20dpf. Gene expression data is presented as relative expression to β-actin, calculated using the formula. 2^-ΔC(t)^. *p<0.050 ^t^p<0.100 (dnMAML vs WT).

The area of non-cartilage and non-osseous tissue, known as the void area within the callus, includes undifferentiated mesenchymal cells, hematopoietic cells, inflammatory cells, and unstained empty space [35]. The percent void space within the callus (Void/TA) was significantly increased in dnMAML mice at 10dpf (Figure 5A). To further characterize this area, semi-quantitative analysis of inflammatory cells was performed at 10dpf, and inflammatory cytokine gene expression was evaluated at 5, 10 and 20dpf. Neutrophil inflammation was characterized as high for dnMAML fractures (>12) and moderate for WT fractures (6–9) (Figure 5B). These values were significantly different. Mononuclear cell inflammation was characterized as mild for both dnMAML and WT fractures (6–9) (Figure 5C). Gram-staining showed no bacterial infection in any fracture (data not shown). Consistent with neutrophil inflammation, Tumor Necrosis Factor-α (TNFα, Figure 5D) and Interleukin-1β (IL-1β, Figure 5E) were upregulated in dnMAML mice at 10dpf. Collectively, the data demonstrates that dnMAML expression results in prolonged inflammatory cell infiltration and cytokine expression during fracture healing.

### dnMAML does not alter cell proliferation during fracture healing

**Figure 6:**
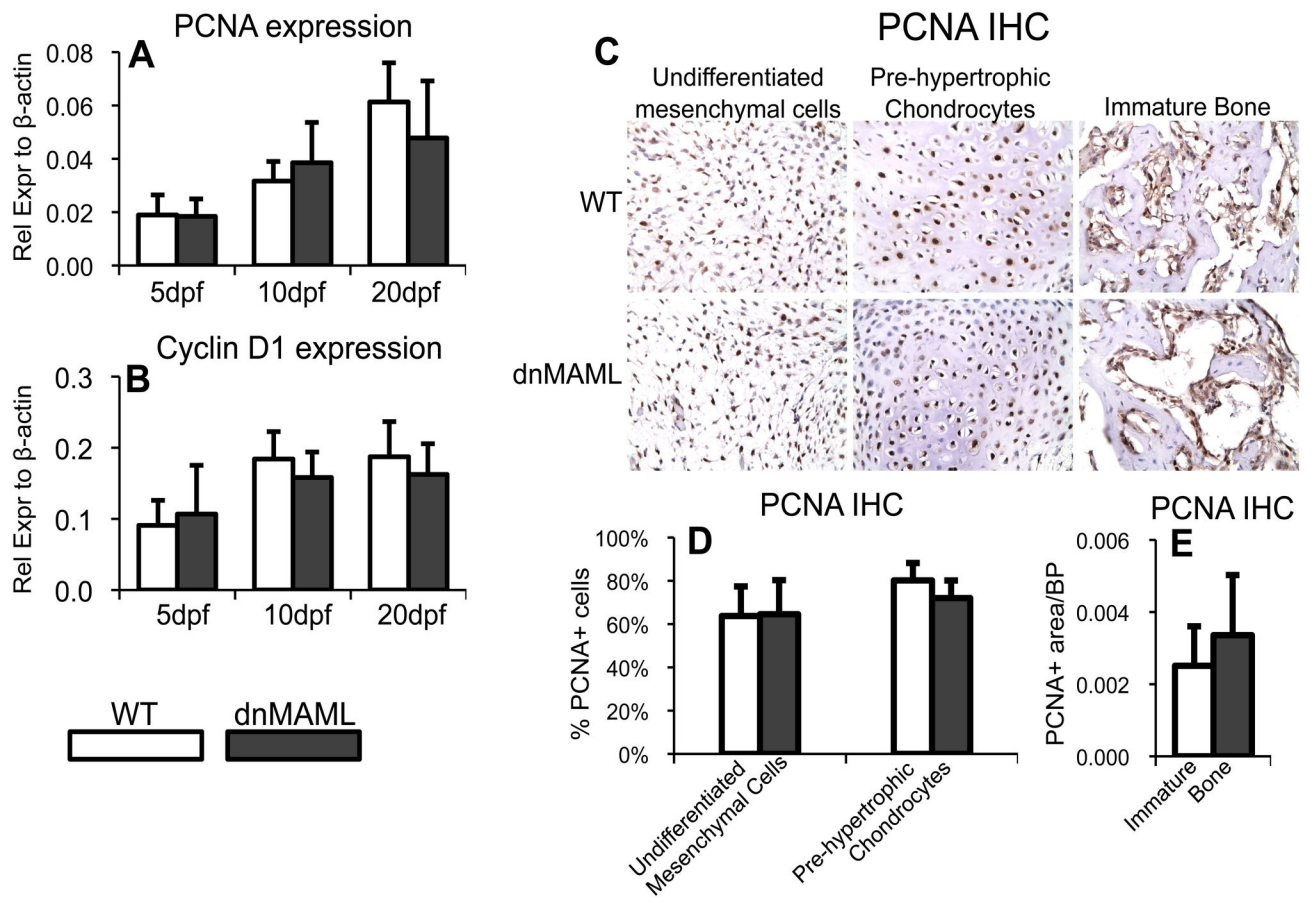
dnMAML does not alter cell proliferation during fracture healing. There are no differences in (**A**) PCNA and (**B**) Cyclin D1, gene expression during fracture healing. (**C**) PCNA IHC staining shows no differences in (**D**) % PCNA+ cells in undifferentiated mesenchymal or in pre-hypertrophic chondrocytes, and no differences in (**E**) PCNA+ area per bone perimeter (PCNA+ area/BP) in immature bone. PCNA IHC images were acquired at 400x magnification. Gene expression data is presented as relative expression to β-actin, calculated using the formula. 2^-ΔC(t)^.

In addition to regulating differentiation, Notch signaling has been shown to control cell proliferation. However, dnMAML expression did not affect proliferation during fracture healing. Specifically, PCNA (Figure 6A) and Cyclin D1 (Figure 6B) gene expression were not different at any of the time points. Furthermore, PCNA IHC staining at 10dpf (Figure 6C) revealed no difference in PCNA staining in undifferentiated mesenchymal cells or pre-hypertrophic chondrocytes (Figure 6D), nor in areas of immature bone formation, which primarily includes osteoblasts, but also endothelial cells, osteoclasts, and other hematopoietic cells (Figure 6E).

### dnMAML inhibits osteoblast differentiation of mMSCs in culture

**Figure 7:**
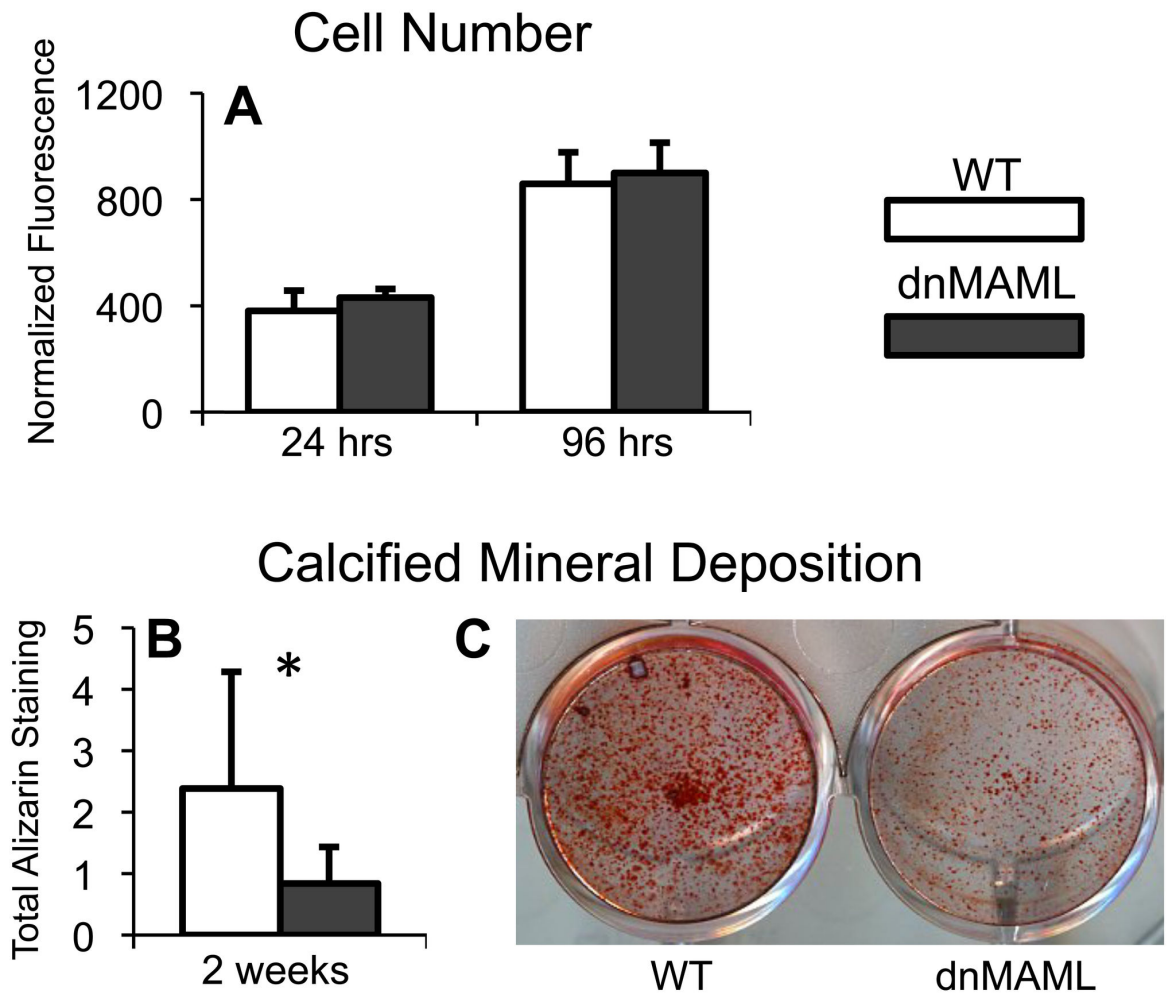
dnMAML inhibits osteoblast differentiation of mMSCs in culture. (**A**) dnMAML does not alter cell number over time (24 hrs p=0.118; 96 hrs p=0.950). (**B**,**C**) dnMAML decreases calcified mineral deposition of differentiated mMSCs (p=0.045).

In fracture healing, the cells that become both bone-forming osteoblasts and chondrocytes that initiate endochondral ossification are referred to as mesenchymal progenitor cells. To better understand the mechanism of Notch signaling in this cell population during fracture healing, bone marrow-derived mMSCs were harvested from uninjured femurs of dnMAML and WT mice. No differences were found in mMSC number at 24 and 96 hours of *in vitro* culture (Figure 7A). However, dnMAML expression decreased calcified mineral deposition of cells (Figure 7B,C), indicating that dnMAML inhibits terminal osteoblast differentiation.

## Discussion

The Notch signaling pathway regulates embryological bone development [7–10,13,14,32,33], and because many aspects of development are recapitulated during repair [17–19], we set out to identify the role of Notch signaling during bone fracture healing. To do this, we crossed heterozygous dnMAML mice with inducible Mx1-Cre promoter mice. A series of poly I:C injections just prior to injury activated the Mx1 promoter and Cre expression in all cell types, resulting in systemic dnMAML expression, which inhibits the Notch signaling pathway at the level of transcriptional complex assembly (NICD-RBPjκ-MAML) just prior to gene transcription [21–24,26]. This protocol has previously been shown to activate dnMAML expression in greater than 95% of total bone marrow cells [25] and 90% of bone marrow-derived mesenchymal progenitor cells [29]. We found that dnMAML activation decreased Hes1 gene expression by 30% at 5dpf. However, we did not observe changes at later time points, as often times analysis of a constantly changing heterogeneous tissue such as the fracture callus more accurately reflects differences in the proportions of cell types present as opposed to changes in gene expression within a homogeneous population on a per cell basis. Furthermore, heterozygous dnMAML mice were chosen as a more clinically relevant model since any potential therapeutic applications that would attempt to inhibit Notch signaling would likely achieve partial but not complete Notch ablation, which may have also contributed to the moderate changes in Notch target gene expression.

Our results demonstrate that Notch signaling is required for the spatiotemporal cascade of healing, where systemic inhibition of canonical Notch signaling alters inflammation, cartilage formation, and bone maturation and remodeling. Our results also indicate that Notch signaling primarily regulates differentiation of the cell-types present in the callus, but doesn’t appear to affect cell proliferation during repair.

The acute inflammatory phase is required to initiate the repair cascade by promoting mesenchymal cell recruitment to the fracture site [36]. However, chronic inflammatory diseases that occur in mouse models such as type I diabetes impair fracture healing [37]. Our results show that systemic Notch inhibition prolongs the inflammatory phase, increasing cytokine gene expression and neutrophil numbers but not mononuclear cell inflammation. Neutrophils and macrophages (a primary component of identifiable mononuclear cells) are the dominant inflammatory cell types present during fracture healing [36,38]. Previous studies have also shown that Notch inhibition prolongs inflammation and delays dermal wound closure [20], results in severe airway inflammation [39], and mice with conditional Notch inhibition in the developing skeleton die prematurely and present with severe ulcerative dermatitis possibly due to excessive inflammation [7]. Collectively, these studies demonstrate the requirement of Notch signaling to resolve the acute inflammatory phase and prevent chronic inflammation.

During endochondral fracture healing, mesenchymal cells recruited to the fracture site condense and undergo chondrogenesis to produce an initial cartilaginous callus [1]. Previous studies have shown that although Notch inhibition enhances chondrogenesis [7,8,10], transient activation of Notch is required to initiate chondrocyte differentiation [12]. In our model, Mx1-Cre mediated systemic Notch inhibition occurred prior to injury, which prevented the transient Notch activation required for chondrogenic induction of mesenchymal cells at the fracture site, and ultimately reduced cartilage formation. Additionally, prolonged inflammation due to Notch inhibition may also be responsible for reduced cartilage formation, as previous studies have shown that inflammatory cytokines inhibit chondrogenesis and chronic inflammation destroys articular cartilage [40–42].

As the cartilaginous callus undergoes resorption, immature bone is produced. Over time, osteoblasts and osteoclasts regulate bone maturation and remodeling. Our results demonstrate that systemic Notch inhibition does not affect early bone formation, and instead alters remodeling during the later stages of repair. The observed increase in BV/TV at 20dpf coincided with a decrease in both total osteogenic cell density (osteoblast and osteocyte) and osteoclast density, suggesting that osteoclast behavior may be the primary downstream regulator responsible for the bone phenotype.

However, the dominant mechanisms regulating the behavior of these cells, whether it is by direct Notch inhibition within a cell type or indirect effects from Notch inhibition in other cell types, is difficult to determine, as the interplay between osteoblasts, osteocytes and osteoclasts is complex. Furthermore, upstream cell types present in the fracture healing cascade, including inflammatory cells and chondrocytes, both of which were altered due to dnMAML expression, can also regulate downstream cell behavior.

For example, our model of systemic Notch inhibition resulted in decreased osteoblast and total osteogenic cell density. With regards to the mechanism regulating osteoblast behavior, we found that during *in vitro* culture, Mx1-Cre mediated dnMAML expression inhibited calcified mineral deposition of bone marrow-derived mMSCs, indicating that Notch inhibition of all mMSCs at all stages of differentiation directly inhibits osteoblast maturation and function. Alternatively, the observed dnMAML osteogenic phenotype may be due to indirect effects of Notch signaling on osteoblasts, as other studies have shown that enhanced expression of inflammatory cytokines secondarily inhibits osteogenesis [43]. Regardless, it is likely that both direct and indirect effects of Notch signaling play an important role in regulating the osteogenic phenotype during fracture healing.

We also observed an increase in the number and proportion of osteocytes in the fracture callus, which could be due to direct effects such as decreased osteocyte apoptosis, and/or indirect effects such as impaired osteoclast-mediated bone resorption and remodeling.

The mechanism regulating osteoclast activity is also complex. Previous studies have shown that Notch inhibition promotes osteoclast differentiation both directly through expression in macrophage precursors [16] and indirectly through expression in osteoblasts and osteocytes [7,13,14]. Expression of inflammatory cytokines has also been shown to promote osteoclast differentiation [44]. However, alternative to this, other studies have shown that an increase in osteocytes via reduced apoptosis inhibits osteoclast-mediated bone resorption [45,46]; and in fact, we observed that systemic dnMAML expression decreases osteoclast density and gene expression, which coincides with an increase in osteocyte density, suggesting that the osteocyte phenotype may in some way regulate osteoclast behavior.

Because of the complexity of the spatiotemporally changing population of cells and tissues during healing, to better understand the direct role of Notch signaling in osteoblasts, osteocytes and osteoclasts, future studies should utilize tissue-specific models of Cre recombinase expression to activate dnMAML in specific cell lineages. Utilizing Prx1, Col3.6, Col2.3, Ocn, or DMP1 promoters would inhibit Notch signaling in undifferentiated mesenchymal progenitors, osteoprogenitors, committed osteoblasts, mature osteoblasts, or osteocytes, respectively. Similarly, TRAP promoters would inhibit Notch signaling in osteoclast lineage cells. Expressing Cre in lineage-restricted inflammatory cells would also be useful for exploring the contribution of inflammatory cells.

Future studies could also utilize gamma secretase inhibitors (GSI), which would allow temporal control of Notch signaling to isolate or exclude it during specific phases of healing. For example, GSI injections following the conclusion of the acute inflammatory phase could exclude any secondary effects of altered inflammation on the rest of healing, providing a model to better understand the direct role of Notch signaling in cartilage formation, callus vascularization, and bone formation and remodeling. Similarly, GSI injections starting at the cartilage-to-bone transition or local delivery of adeno-Cre would isolate the role of Notch signaling during bone formation and remodeling.

In conclusion, our results demonstrate that the Notch signaling pathway is required for the proper temporal cascade of bone fracture healing, and that systemic inhibition of the pathway for the duration of healing is not an ideal therapeutic to improve regeneration. However, more research is required to understand the role of Notch signaling in individual cell populations during repair.

## Supporting Information

Figure S1 Sample size for each group per time point per assay. (DOCX)

Figure S2 Primer list for each gene analyzed including forward and reverse sequence with accession number. (DOCX)

Figure S3 Summary of statistical analyses. (**A**) For gene expression (data normalized to β-actin), histology (at 20x magnification), and μCT analysis, a two-way ANOVA was conducted to evaluate the effects of time, dnMAML expression, and the interaction, with a post-hoc student’s t-test comparing dnMAML to WT at each time point when the effect of dnMAML expression or the interaction was significant or a trend. (**B**) For cell- and tissue-specific histomorphometric analysis (at 200x and 400x magnification) and Notch gene expression analysis (data normalized to WT control for each time point), a student’s t-test was used to compare dnMAML to WT at each time point. (**C**) For semi-quantitative analysis of inflammation, a Mann-Whitney U non-parametric test was used to compare dnMAML to WT. Significance was set at p<0.050 (*) and a trend at p<0.100 (^t^) (n/a). means that statistical test was not run because ANOVA was non-significant. (-) means that no statistical test was run because no data set was collected at that time point.
(DOCX)
