# Supplementary material for: Systemic Inhibition of Canonical Notch Signaling Results in Sustained Callus Inflammation and Alters Multiple Phases of Fracture Healing
Source: PLoS One. 2013 Jul 3;8(7):e68726. doi: 10.1371/journal.pone.0068726 (PMC3701065; doi:10.1371/journal.pone.0068726)
Supplement: Figure S1 — (DOCX) [file pone.0068726.s001.docx]

| Assay | Group | 5dpf | 10dpf | 20dpf |
| --- | --- | --- | --- | --- |
| RT-PCR | dnMAML | 6 | 8 | 9 |
|  | WT | 6 | 9 | 8 |
| Histology | dnMAML | - | 6 | 4 |
|  | WT | - | 7 | 4 |
| μCT | dnMAML | - | 7 | 13 |
|  | WT | - | 8 | 9 |
