# Supplementary material for: Systemic Inhibition of Canonical Notch Signaling Results in Sustained Callus Inflammation and Alters Multiple Phases of Fracture Healing
Source: PLoS One. 2013 Jul 3;8(7):e68726. doi: 10.1371/journal.pone.0068726 (PMC3701065; doi:10.1371/journal.pone.0068726)
Supplement: Figure S2 — (DOCX) [file pone.0068726.s002.docx]

| Gene | Forward Primer | Reverse Primer | Accession # |
| --- | --- | --- | --- |
| Bactin | AAGAGCTATGAGCTGCCTGA | TGGCATAGAGGTCTTTACGG | [NM_007393](http://www.ncbi.nlm.nih.gov/entrez/viewer.fcgi?db=nucleotide&id=145966868) |
| Hes1 | CCAAGCTAGAGAAGGCAGACA | GTCACCTCGTTCATGGACTC | [NM_008235](http://www.ncbi.nlm.nih.gov/entrez/viewer.fcgi?db=nucleotide&id=31560817) |
| Col2a1 | GGCTCCCAGAACATCACCTA | TCGGCCCTCATCTCTACATC | NM_031163 |
| Sox9 | AAGTTCCCCGTGTGCATC | GCCTGCGCCCACAC | NM_011448 |
| ColX | CGTGTCTGCTTTTACTGTCA | ACCTGGTCATTTTCTGTGAG | NM_009925 |
| Ocn | CGCTCTGTCTCTCTGACCTC | TCACAAGCAGGGTTAAGCTC | NM_007541 |
| Osx | TCTCTCCATCTGCCTGACTC | GTCAGCGTATGGCTTCTTTG | NM_178770 |
| Col1a1 | AATGGTGCTCCTGGTATTGC | GGCACCAGTGTCTCCTTTGT | NM_007742 |
| TRAP | CGGTACAGCCCCCACTCCCA | CGCTGGCATCGTGCACTCCA | NM_001102405 |
| IL-1B | GCCCATCCTCTGTGACTCAT | AGGCCACAGGTATTTTGTCG | NM_008361 |
| TNF-a | CTGCCGTCAAGAGCCCCTGC | AGCGCTGAGTTGGTCCCCCT | NM_013693 |
| PCNA | CCACATTGGAGATGCTGTTG | CAGTGGAGTGGCTTTTGTGA | NM_011045 |
| CyclinD1 | AGTGCGTGCAGAAGGAGATT | CACAACTTCTCGGCAGTCAA | [NM_007631](http://www.ncbi.nlm.nih.gov/entrez/viewer.fcgi?db=nucleotide&id=119672895) |
| GFP | GACGACGGCAACTACAAGAC | TCGGCCATGATATAGACGTT |  |
