# Supplementary material for: Systemic Inhibition of Canonical Notch Signaling Results in Sustained Callus Inflammation and Alters Multiple Phases of Fracture Healing
Source: PLoS One. 2013 Jul 3;8(7):e68726. doi: 10.1371/journal.pone.0068726 (PMC3701065; doi:10.1371/journal.pone.0068726)
Supplement: Figure S3 — (A) For gene expression (data normalized to β-actin), histology (at 20x magnification), and μCT analysis, a two-way ANOVA was conducted to evaluate the effects of time, dnMAML expression, and the interaction, with a post-hoc student’s t-test comparing dnMAML to WT at each time point when the effect of dnMAML expression or the interaction was significant or a trend. (B) For cell- and tissue-specific histomorphometric analysis (at 200x and 400x magnification) and Notch gene expression analysis (data normalized to WT control for each time point), a student’s t-test was used to compare dnMAML to WT at each time point. (C) For semi-quantitative analysis of inflammation, a Mann-Whitney U non-parametric test was used to compare dnMAML to WT. Significance was set at p<0.050 (*) and a trend at p<0.100 (t) (n/a). means that statistical test was not run because ANOVA was non-significant. (-) means that no statistical test was run because no data set was collected at that time point. (DOCX) [file pone.0068726.s003.docx]

| A | Parameter | Figure/Table | Two-Way ANOVA | | | Post-Hoc Student’s t-test | | |
| --- | --- | --- | --- | --- | --- | --- | --- | --- |
|  |  |  | Time | dnMAML | Interaction | 5dpf | 10dpf | 20dpf |
|  | Gene Expression | | | | | | | |
|  | Col2a1 | Figure 2B | 0.003* | 0.005* | 0.069^t^ | 0.409 | 0.086^t^ | 0.498 |
|  | Sox9 | Figure 2C | 0.019* | 0.004* | 0.071^t^ | 0.406 | 0.078^t^ | 0.269 |
|  | ColX | Figure 2D | 0.005* | 0.028* | 0.204 | 0.538 | 0.182 | 0.656 |
|  | Ocn | Figure 4E | 0.000* | 0.937 | 0.033* | 0.269 | 0.866 | 0.048* |
|  | Osx | Figure 4F | 0.000* | 0.367 | 0.817 | n/a | n/a | n/a |
|  | Col1a1 | Figure 4G | 0.000* | 0.990 | 0.887 | n/a | n/a | n/a |
|  | TRAP | Figure 4I | 0.000* | 0.767 | 0.058^t^ | 0.253 | 0.742 | 0.046* |
|  | TNF-a | Figure 5D | 0.118 | 0.009* | 0.369 | 0.533 | 0.027* | 0.179 |
|  | IL-1B | Figure 5E | 0.045* | 0.005* | 0.027* | 0.284 | 0.073^t^ | 0.010* |
|  | PCNA | Figure 6A | 0.001* | 0.319 | 0.120 | n/a | n/a | n/a |
|  | CyclinD1 | Figure 6B | 0.054^t^ | 0.247 | 0.408 | n/a | n/a | n/a |
|  | Histology | | | | | | | |
|  | CA/TA | Figure 2A | 0.000* | 0.002* | 0.016 | - | 0.010* | 0.331 |
|  | BA/TA | Figure 3B | 0.005* | 0.405 | 0.506 | - | n/a | n/a |
|  | Avg TA | Figure 3E | 0.004* | 0.120 | 0.019* | - | 0.133 | 0.089^t^ |
|  | Avg BA | Figure 3F | 0.928 | 0.107 | 0.141 | - | n/a | n/a |
|  | μCT | | | | | | | |
|  | BV/TV | Figure 3A | 0.000* | 0.753 | 0.010* | - | 0.442 | 0.006* |
|  | TV | Figure 3C | 0.018* | 0.496 | 0.116 | - | n/a | n/a |
|  | BV | Figure 3D | 0.000* | 0.879 | 0.487 | - | n/a | n/a |
|  | Tb.N | Table 1 | 0.000* | 0.701 | 0.149 | - | n/a | n/a |
|  | Tb.Th | Table 1 | 0.019* | 0.984 | 0.020* | - | 0.968 | 0.004* |
|  | Tb.Sp | Table 1 | 0.000* | 0.235 | 0.295 | - | n/a | n/a |
|  | TMD | Table 1 | 0.000* | 0.862 | 0.130 | - | n/a | n/a |
|  | SMI | Table 1 | 0.000* | 0.747 | 0.027* | - | 0.554 | 0.015* |
|  | Conn.D | Table 1 | 0.000* | 0.557 | 0.098^t^ | - | 0.255 | 0.015* |

| B | Parameter | Figure/Table | Student’s t-test | | |
| --- | --- | --- | --- | --- | --- |
|  |  |  | 5dpf | 10dpf | 20dpf |
|  | Cartilage Histomorphometry | | | | |
|  | Immature Cartilage/CA | Figure 2E | - | 0.637 | - |
|  | Mature Cartilage/CA | Figure 2E | - | 0.901 | - |
|  | Hypertrophic Cartilage/CA | Figure 2E | - | 0.404 | - |
|  | Proliferating Chondrocyte Density | Figure 2F | - | 0.560 | - |
|  | Pre-hypertrophic Chondrocyte Density | Figure 2F | - | 0.261 | - |
|  | Hypertrophic Chondrocyte Density | Figure 2F | - | 0.378 | - |
|  | Bone Histomorphometry | | | | |
|  | Osteoblast Density | Figure 4A | - | 0.577 | 0.005* |
|  | Osteocyte Density | Figure 4B | - | 0.174 | 0.022* |
|  | Osteocyte:Osteoblast Ratio | Figure 4C | - | 0.221 | 0.000* |
|  | Total Osteogenic Cell Density | Figure 4D | - | 0.604 | 0.011* |
|  | Osteoclast Density | Figure 4H | - | 0.741 | 0.029* |
|  | % PCNA+ Cells or Area | | | | |
|  | Undifferentiated Mesenchymal | Figure 6D | - | 0.934 | - |
|  | Pre-hypertrophic Chondrocytes | Figure 6D | - | 0.150 | - |
|  | Immature Bone | Figure 6E | - | 0.369 | - |
|  | Inflammation | | | | |
|  | Void/TA | Figure 5A | - | 0.040* | - |
|  | Notch Gene Expression | | | | |
|  | GFP | Figure 1A | 0.000* | 0.000* | 0.000* |
|  | Hes1 | Figure 1B | 0.026* | 0.202 | 0.976 |

| C | Parameter | Figure/Table | Mann-Whitney U test: 10dpf |
| --- | --- | --- | --- |
|  | Inflammation | | |
|  | Neutrophil | Figure 5B | 0.005* |
|  | Mononuclear | Figure 5C | 0.302 |
